# Multi-payload antibody-drug conjugates for photothermal dynamic and chemotherapy of HER2-positive breast cancer

**DOI:** 10.1016/j.mtbio.2026.103355

**Published:** 2026-06-12

**Authors:** Yuqi Zhang, Yongxiang Bai, Xingxiang Ren, Yurong Fan, Zhengzhong Lv, Qingfei Song, Xiaoyan Wang, Haibin Shi

**Affiliations:** a State Key Laboratory of Radiation Medicine and Protection, School of Radiation Medicine and Protection, Collaborative Innovation Center of Radiological Medicine of Jiangsu Higher Education Institutions, Soochow University, Suzhou, 215123, China; b Department of Ultrasound, Heping Hospital Affiliated to Changzhi Medical College, Changzhi, 046000, China

**Keywords:** Antibody-drug conjugates, Photothermal-thermodynamic therapy (PTDT), Chemotherapy, HER2-Positive breast cancer

## Abstract

HER2-positive breast cancer is highly aggressive and has a poor prognosis in clinical treatment primarily due to the overexpression of HER2 protein. Trastuzumab can significantly improve the therapeutic outcome for HER2-positive breast cancer, but 70% of patients develop drug resistance. Trastuzumab-drug conjugates have emerged as a promising modality in the treatment of HER2-positive breast cancer, while their therapeutic efficacy remains limited. Herein, we report a multi-payload trastuzumab conjugate, QC-AA@Tra, prepared by attaching an AIPH-bearing NIR-II fluorophore (Q3) to trastuzumab via nucleophilic substitution. After intravenous administration, QC-AA@Tra selectively accumulates at tumor sites. Under 808 nm laser irradiation, it produces a strong photothermal–dynamic effect through thermal release of nitrogen gas and free radicals from AIPH. Combined with trastuzumab's targeting, this leads to marked suppression of HER2-positive BT474 xenograft tumors in mice. Our design may offer a powerful and universal modality for precise diagnosis and treatment of HER2-positive malignant tumors.

## Graphical abstract

Leveraging a three-site NIR-II scaffold–enabled multi-payload antibody–drug conjugation strategy, a trastuzumab-based therapeutic platform was developed to integrate NIR-II fluorescence imaging with laser-triggered photothermal dynamic therapy and chemotherapy, enabling precise tumor targeting and potent suppression of HER2-positive breast tumors in vivo.

**Figure:**
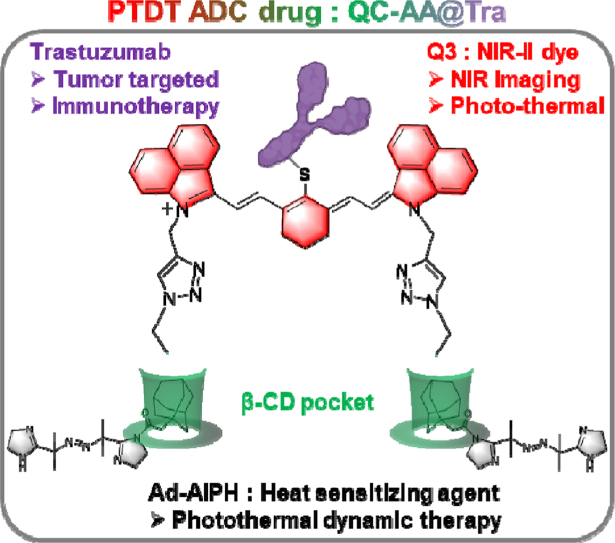

## 1 Introduction

Breast cancer is the most common type of malignant tumor worldwide and a leading cause of cancer death among women due to poor prognosis. Approximately 20-30% of breast cancers overexpress HER2 protein, making them more aggressive and faster-growing than other types [[1], [2], [3], [4]]. Trastuzumab (Tra), a monoclonal antibody that specifically targets the HER2 protein, has revolutionized the treatment of HER2-positive breast cancer by inhibiting cancer cell growth and improving patient prognosis [[5], [6], [7], [8]]. However, about 70% of breast cancer patients develop primary or secondary resistance to trastuzumab treatment, posing a huge clinical challenge [[9], [10], [11], [12], [13], [14]]. Recent studies have proven that antibody-drug conjugates (ADCs), such as T-DXd and T-DM1, are highly effective for the treatment of patients with HER2-positive breast cancer, whereas there is about 21% of patients with poor tumor suppression or remission [[15], [16], [17]]. Therefore, developing advanced and efficient trastuzumab-based ADCs is of great significance for clinical treatment of HER2-positive malignant breast tumor.

Photothermal-thermodynamic therapy (PTDT) that integrates photothermal and chemodynamic therapies into a single system by simultaneously converting near-infrared (NIR) light into heat and reactive oxygen species (ROS) has recently attracted tremendous attention for solid tumor treatment [[18], [19], [20], [21], [22], [23]]. PTDT has the advantage over traditional photodynamic therapy (PDT) in that it is effective in generating free radicals even in the hypoxic tumor microenvironment. However, the commonly used PTDT agents are mostly nanomaterials that have potential metabolic and safety risks. Therefore, the development of efficient PTDT agents based on tumor-targeted therapeutic antibodies is highly expected to improve the anti-tumor effect while having low side effect. In our previous studies, NIR-II dye ZM1068 has been found to exhibit excellent photothermal property and is a potential PTDT formulation [[24], [25], [26]]. Notably, the unique chloro group of ZM1068 can readily undergo nucleophilic substitution reaction with the thiol groups of proteins under mild condition, making it an excellent connector for ADC drug construction.

**Fig. 1:**
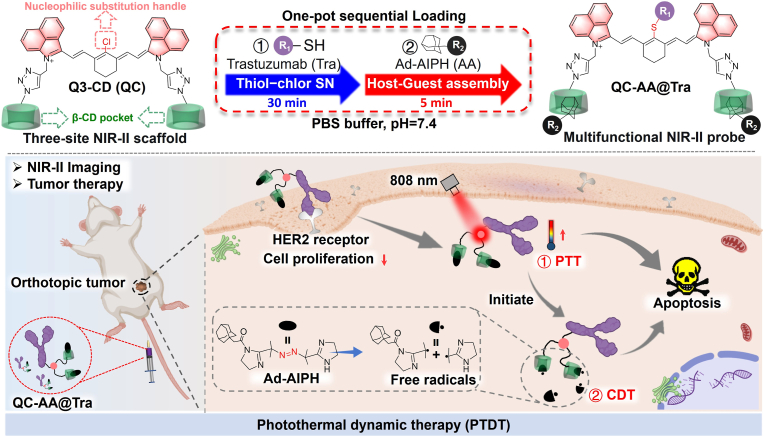
Schematic diagram of multi-payload antibody-drug conjugate-based therapeutic platform integrating PTDT and chemotherapy for the treatment of HER2-positive breast cancer.

In this study, we present a novel multi-payload Trastuzumab-drug conjugate (QC-AA@Tra) for synergistic photothermal dynamic therapy and chemotherapy of HER2-positive breast tumors. QC-AA@Tra was rationally designed and constructed by conjugating a β-cyclodextrin (β-CD)-modified NIR-II fluorophore Q3 onto trastuzumab via a thiol-chlor nucleophilic substitution reaction (thiol-chlor SN), followed by encapsulation of the thermosensitive agent Ad-AIPH into β-CD through host–guest inclusion and hydrophobic interactions [[27], [28], [29], [30], [31], [32]]. As illuminated in Fig. 1, QC-AA@Tra can selectively target and be internalized by HER2-overexpressing cancer cells through the specific binding of trastuzumab to HER2 receptor [[33], [34], [35], [36]]. Taking advantage of intensive NIR-II fluorescence and the thermal-dependent N_2_ and free radicals release of QC-AA@Tra under 808 nm laser irradiation, significant suppression of HER2-positive BT474 xenograft tumors was achieved in living mice by fluorescence imaging-guided synergistic PTDT and chemotherapy.

## 2 Experimental section

### 2.1 Materials

Mono-6-deoxy-6-azido-β-cyclodextrin, CuSO_4_·5H_2_O, sodium ascorbate, 1-adamantaneacetic acid, oxalyl dichloride, 2,2′-azobis[2-(2-imidazolin-2-yl) propane] dihydrochloride, a-(4-Pyridyl N-oxide)-N-tert-butylnitrone, POBN (tert-butyl-[(1-oxidopyridin-4-ylidene)methyl]-oxoazanium) and DCFH-DA were purchased from Saan chemical technology (Shanghai) CO., Ltd. Trastuzumab, Anti-Trastuzumab, Goat Anti-Rabbit IgG (H + L) FITC were bought from Abways technology, Inc. BT474 and HEK293 cells were provided by Shanghai Fuheng Biotechnology Co., Ltd. CCK8 and Calcein/PI cell activity and cytotoxicity assay kit and Annexin V-FITC/Pl Apoptosis Kit were purchased from Beyotime Biotechnology Co., Ltd.

### 2.2 Characterization

The absorption spectra were measured on an ultraviolet-visible-near-infrared UV-3600 (UV-vis-NIR) spectrophotometer (Shimadzu, Kyoto, Japan). The FL spectrum was determined on an FLS980 spectrometer (Edinburgh Instruments, UK). HPLC analysis was performed with an Agilent 1260 high-performance liquid chromatography system. In vivo fluorescence images were obtained using the IVIS spectrum imaging system (PerkinElmer) and the NIR-II imaging system from Suzhou Yingrui Optical Technology Co., LTD. Confocal microscopy images were captured using an Olympus FV1200 fluorescence microscope. All data were analyzed and calculated with Microsoft Excel 2019 software (Microsoft), and the statistical differences were analyzed by a two-tailed student's test.

### 2.3 Synthesis of Q3-CD

NIR-II fluorophore Q3 was synthesized according to the previous reported method. Compound Q3 (5.47 mg, 0.01 mmol) was dissolved in dimethyl sulfoxide (5.0 mL) with constant stirring followed by the addition of mono-6-deoxy-6-azido-β-cyclodextrin (34.8 mg, 0.03 mmol), CuSO_4_·5H_2_O (7.49 mg, 0.03 mmol), sodium ascorbate (5.94 mg, 0.03 mmol), and 1 mL H_2_O. The mixture was subsequently stirred at room temperature for 1 h. Then the solution was concentrated under reduced pressure to yield black solid which was purified by HPLC to yield pure Q3-CD as dark solid (15.7 mg, 55%). ^1^H NMR (600 MHz, DMSO-*d*_6_*)* δ 8.68 (d, *J* = 13.4 Hz, 1H), 8.38 (d, *J* = 7.6 Hz, 1H), 8.25–8.15 (m, 1H), 7.98–7.86 (m, 1H), 7.79–7.54 (m, 2H), 7.24–6.88 (m, 2H), 5.65 (s, 6H), 4.80 (d, *J* = 30.5 Hz, 4H), 3.97–3.08 (m, 33H), 2.95 (dd, *J* = 84.5, 8.4 Hz, 3H), 1.27–1.16 (m, 1H). MALDI-MS: Calcd. For C_122_H_166_ClN_8_O_68_^+^ [M^+^] 2866.9494, found: 2867.7713.

### 2.4 Synthesis of Ad-AIPH

1-Adamantaneacetic acid (194.3 mg, 1 mmol) and oxalyl dichloride (126.9 mg, 1 mmol) were dissolved in dichloromethane and stirred for 2 h. Then the solution was concentrated under reduced pressure followed by addition of 2,2′-azobis[2-(2-imidazolin-2-yl)propane]dihydrochloride (969.81 mg, 3 mmol) and 5 mL dichloromethane for 2 h stirring. The solution was then concentrated under reduced pressure to yield white solid after purification by HPLC. Pure Ad-AIPH was collected as white solid (375.2 mg, 88%). ^1^H NMR (600 MHz, CDCl_3_) δ 4.76 (s, 1H), 4.32 – 4.21 (m, 1H), 3.89 (s, 2H), 3.79 – 3.72 (m, 2H), 2.60 (d, *J* = 19.6 Hz, 2H), 1.48 (t, *J* = 7.0 Hz, 1H), 1.42 (s, 1H), 1.37 (s, 1H), 1.28 (dt, *J* = 32.3, 13.7 Hz, 8H). ^13^C NMR (151 MHz, CDCl3) δ 107.52, 107.43, 100.17, 99.89, 66.62, 57.40, 55.95, 53.43, 48.82, 40.26, 31.91, 31.49, 31.42, 30.30, 30.17, 30.12, 30.04, 29.69, 29.35, 22.68, 14.74. MALDI-MS: Calcd. For C_24_H_38_N_6_O [M] 426.3107, found: 427.6230.

### 2.5 Preparation of QC@Tra

1 mg of trastuzumab was dissolved in 1 mL of PBS buffer and then added 0.79 mg of Q3-CD followed by thorough mixing. The mixture was shaking for 30 min at 25°C. Finally, the mixture was washed three times with PBS buffer and purified by a 50 kDa ultrafiltration tube to obtain QC@Tra (200 μL, 34.5 μM).

### 2.6 Preparation of QC-AA@Tra and QC-AA

QC@Tra (100 μL, 34.5 μM) and 29 μg of Ad-AIPH was added under ultrasonic condition and continued for 5 min. Finally, the resulting mixture was subjected to low-speed centrifugation at 500 rpm for 5 min to remove insoluble precipitates affording QC-AA@Tra (100 μL, 34.5 μM). Q3-CD (100 μL, 34.5 μM) and 29 μg of Ad-AIPH was added under ultrasonic condition and continued for 5 min. Finally, the resulting mixture was subjected to low-speed centrifugation at 500 rpm for 5 min to remove insoluble precipitates affording QC-AA (100 μL, 34.5 μM).

### 2.7 Photothermal dynamic effect of QC-AA@Tra

Various concentrations of 250 μL QC-AA@Tra (0, 20, 40, 80, and 160 μg/mL) were irradiated by an 808 nm (1 W/cm^2^) laser for 10 min. The infrared images and temperature curves were recorded using a thermal imaging camera. To monitor the release of free radicals, 2 mL of QC-AA@Tra (160 μg/mL) solution was mixed with 100 mM of POBN (tert-butyl-[(1-oxidopyridin-4-ylidene)methyl]-oxoazanium) solution in a vial followed by 808 nm irradiation (1 W/cm^2^). 200 μL of the solution was then transferred into a quartz capillary tube and then determined by an ESR spectrometer at 0, 2, 5, and 10 min. The photothermal conversion efficiency (*η*) was calculated using the following equation (1).

(1) $$\eta =\frac{m\;\cdot \;c\;\cdot \;\left(T_{\max}-{\;T}_{{\max\;\cdot \;H}_{2}O}\right)}{I\;\cdot \;\left(1-10^{-A}\;\right)\;\cdot {\;\tau }_{S}}$$

where m is the solution mass, c is the heat capacity of water (4.2 J/g), T_max_ and T_max·H2O_ are maximum temperatures achieved in the presence or absence of QC-AA@Tra, respectively. I is the laser power density (1 W cm^−2^), A is the absorbance of the aqueous solutions of QC-AA@Tra at 808 nm, and τ_s_ is system time constant.

### 2.8 Cellular uptake and in vitro targeting ability

BT474 and HEK293 cells were seeded in confocal culture dishes and cultured for 24 h. 1 μM of QC-AA@Tra was added and cultured for 0, 0.5, 2, 4, and 8 h. The cells were then washed three times with PBS buffers, and then incubated with FITC-labeled secondary antibody (Anti-trastuzumab) for 2 h. The cells were eventually observed under a confocal laser scanning microscope (CLSM). After that, the cells were collected for flow cytometry analysis.

### 2.9 Cytotoxicity assays

The cytotoxicity of the probes toward BT474 and HEK293 cells was tested using the CCK-8 assay. In brief, the cells were seeded into 96-well plate and grew for 24 h. The culture medium was then removed, and the probes were added into the cells followed by 24 h incubation. After that, the medium was removed, and the cells were washed three times with PBS. CCK-8 reagent was subsequently added into the cells and incubated for further 2 h. The absorbance of each well was measured at 450 nm using a microplate reader. Besides, the cells were exposed to 808 nm laser light (1 W/cm^2^) for 5 min and then placed back in the incubator for an additional 24 h incubation before cytotoxicity was assessed using the CCK-8 assay. The half-maximal inhibitory concentration (IC50) was calculated by fitting the concentration-response data using a four-parameter logistic nonlinear regression model in OriginPro 2026 using the following formula (2):

(2) $$y=b+\frac{a-b}{1+{\left(\;\frac{x}{c}\;\right)}^{d}\;}$$

Where y is the mortality percentage and x is the concentration. The four parameters represent: (a) the minimum value (at zero dose), (b) the maximum value (at infinite dose), (c) the inflection point, and (d) the Hill's slope, which describes the steepness of the curve at the inflection point.

### 2.10 Live/dead cell assay

BT474 and HEK293 cells were seeded into 6-well plates and cultured for 24 h. The cells were then incubated with 1 μM QC-AA@Tra for an additional 24 h. The culture medium was removed and replaced with fresh medium followed by exposure of an 808 nm laser (1 W/cm^2^) for 0, 5, and 10 min. After 24 h, 1 mL of Calcein AM/PI detection solution was added to each well. The cells were incubated at 37°C in the dark for 30 min. Finally, the staining results were observed under a fluorescence microscope (Calcein AM: *E*_*x*_/*E*_*m*_ = 494/517 nm; PI: *E*_*x*_/*E*_*m*_ = 535/617 nm).

### 2.11 Detection of intracellular oxidative stress

BT474 cells and HEK293 were seeded into confocal culture dishes and incubated for 24 h. The cells were then incubated with 1 μM QC-AA@Tra for 0, 2, 4, and 8 h. After that, the cells were washed three times with PBS buffer. Next, 1 mL of DCFH-DA (10 μM) was added into each dish, and the cells were incubated at 37°C for 20 min. The cells were subsequently washed three times with serum-free cell culture medium to remove DCFH-DA that was not internalized by cells. The cells were further irradiated by an 808 nm laser (1 W/cm^2^) for 5 min and imaged using a laser confocal microscope.

### 2.12 Apoptosis detection assays

BT474 cells were seeded into 6-well plates and cultured for 24 h. Subsequently, the cells were incubated with 5 μM of QC-AA@Tra or QC@Tra for 24 h. After that, the medium was replaced by fresh culture medium, and the cells were exposed to an 808 nm laser (0.3 W/cm^2^) for 0, 1, 2 and 4 min. After 24 h incubation, the cells were trypsinized, collected by centrifugation, and washed once with PBS. The cells were gently resuspended and counted. A total of 5 × 10^5^ cells were centrifuged, and the supernatant was discarded. The cells were then resuspended in 500 μL of diluted 1 × Annexin V Binding Buffer. To this suspension, 5 μL of Annexin V-FITC reagent and 5 μL of PI reagent (50 μg/mL) were added. The mixture was gently vortexed and incubated in the dark at room temperature for 20 min. The samples were immediately analyzed by flow cytometry.

### 2.13 Animals

All animal experiments were conducted according to the Guidelines for the Care and Use of Laboratory Animals of Soochow University and were approved by the Animal Ethics Committee of the Soochow University Laboratory Animal Center (Suzhou, China). In the sub-cutaneous tumor model, tumor weight was restricted to under 10% of normal body weight or maximum diameter of 13 mm (approximately 1098.5 mm^3^ in volume). Six-week-old Balbc-Nu female mice, weighing 14-16 g, were obtained from GemPharmatech Co., Ltd. The mice were kept in standard conditions with a temperature of 25 ± 3°C, relative humidity of 60% ± 10%, and a 12 h light/dark cycle. Tumors were induced by injecting 3 × 10^6^ BT474 cells suspended in 50 μL of PBS into the breast of each mouse, and the tumor formation rate was 67.5%. Tumor dimensions were measured using calipers, and volumes were calculated with the formula (length×width^2^)/2. Throughout all experiments, tumor volumes were main-tained within the limits set by the Animal Ethics Committee of Soochow University (Ethics approval number: 202411A1016). In the subcutaneous tumor model, tumor weight was restricted to under 10% of normal body weight or maximum diameter of 13.4 mm (approximately 1200 mm^3^ in volume).

### 2.14 NIR-II imaging of breast cancer in living mice

QC-AA or QC-AA@Tra (10 mg/kg) was administered in BT474 tumor-bearing mice through the tail vein. Real-time NIR-II imaging was subsequently performed on the NIR-II imaging system (excitation: 808 nm, emission: 1000 nm). The average signal intensity was calculated using Image J software at three different points. NIR-II fluorescence imaging of major organs was carried out at 8 h post-injection. The average signal intensity was calculated using Image J software at three different points.

### 2.15 Photothermal dynamic and chemotherapy

Nude mice with BT474 xenograft tumors were randomly divided into several groups with four mice per group. Each mouse received intravenous injection of 200 μL PBS buffer or 10 mg/kg QC-AA@Tra. After 8 h, the tumors were irradiated by an 808 nm laser (1 W/cm^2^) for 5 min. The tumor size and body weight were measured every two days. On the 20th day, the mice were sacrificed for further analysis. The tumor inhibition rate (TIR) was calculated on day 20 after sacrifice and tumor resection using the following formula (3):

(3) $$\text{TIR}\;\left(\%\right)=\frac{{V_{\text{contro}l}-V}_{\text{treated}}}{V_{\text{contro}l}}\times 100\%$$

where V_control_ is the mean tumor volume of the negative control group (PBS treated mice), and V_treated_ is the mean tumor volume of the experimental group (QC-AA@Tra +808 nm).

## 3 Results and discussion

### 3.1 Design, synthesis and characterization of QC-AA@Tra

The study began with the synthesis of probe QC-AA@Tra. NIR-II dye Q3 was synthesized according to the method previously reported [37,38]. Two mono-6-deoxy-6-azido-β-cyclodextrin were subsequently incorporated onto Q3 through the azide-alkyne click chemistry to yield Q3-CD [39]. Next, Q3-CD was conjugated onto trastuzumab via the nucleophilic substitution reaction between thiol and chloro group to give QC@Tra. An adamantane-containing thermal sensitizer Ad-AIPH was then synthesized and encapsulated into QC@Tra through the inclusion and hydrophobic interaction to afford multi-payload trastuzumab conjugate QC-AA@Tra (Scheme S1 and S2). All intermediates were confirmed by mass spectrometry (Fig. S1–S6).

**Fig. 2:**
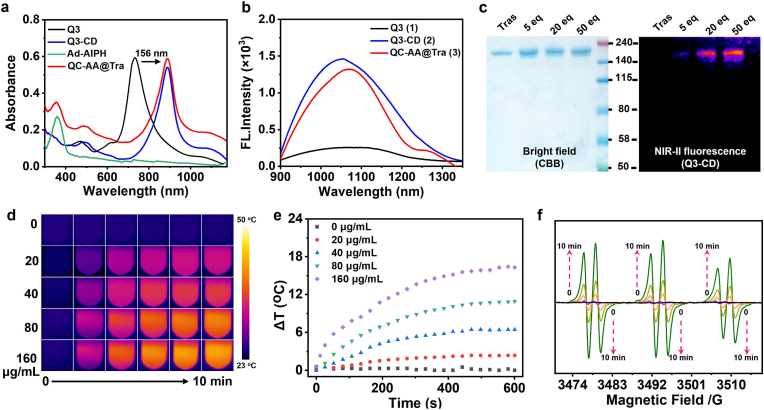
(a) Absorption spectra of Q3, Q3-CD, Ad-AIPH and QC-AA@Tra aqueous solutions. (b) Emission spectra of Q3, Q3-CD, and QC-AA@Tra aqueous solutions. (c) CBB (Coomassie Brilliant Blue) staining and NIR-II fluorescence images of the reactions of trastuzumab (0.1 mg) with various amounts of Q3-CD (0 μg/24.7 μg/98.9 μg/247.2 μg) in PBS buffer (100 μL). (d) Photothermal images of different concentrations of QC-AA@Tra upon 808 nm laser irradiation (1 W/cm^2^). (e) The temperature change curve in d. (f) ESR spectra of POBN (100 mM) treated with 160 μg mL^−1^ of QC-AA@Tra for various irradiation times (808 nm, 1 W cm^−2^). The arrows indicate the spectral changes with increasing irradiation time.

We next examined the absorption spectra of Q3, Ad-AIPH, Q3-CD, and QC-AA@Tra. Fig. 2a indicates Q3-CD and QC-AA@Tra have a similar absorption spectrum in the near-infrared region and exhibit an obvious absorbance maximum at 889 nm with a red shift of approximately 156 nm compared to Q3, which should be attributed to the increased water solubility due to the presence of cyclodextrin. However, a significantly enhanced absorption peak at 350 nm was determined only for QC-AA@Tra implying that Ad-AIPH has been successfully loaded. The fluorescence spectra show that Q3-CD and QC-AA@Tra emit strong NIR-II fluorescence, and the introduction of cyclodextrin greatly enhances the molecule's fluorescence signals in aqueous phase (Fig. 2b), which is highly beneficial for subsequent bioimaging application. To evaluate whether Q3-CD can be conjugated onto trastuzumab, we next analyzed the assays of different ratios of Q3-CD to trastuzumab through running polyacrylamide gel electrophoresis (Fig. 2c). As the molar ratio of Q3-CD to trastuzumab increased, the labeling efficiency of trastuzumab by Q3-CD was gradually increased, strongly supporting that trastuzumab can be efficiently labeled by Q3-CD at ambient condition. Hence we chose 20:1 M ratio of trastuzumab to Q3-CD to construct QC-AA@Tra for subsequent experiments.

Considering strong absorption of the probes in near infrared region, we further studied the photothermal effect of QC-AA@Tra in aqueous solution. Various concentrations of QC-AA@Tra aqueous solutions were exposed to an 808 nm laser (1 W cm^−2^) for 10 min, and their temperatures were monitored using an infrared camera. As shown in Fig. 2d and e, the temperature of the QC-AA@Tra solutions increased progressively with the increase of irradiation time and the concentrations from 0 to 160 μg mL^−1^. The photothermal conversion efficiency (η%) of QC-AA@Tra was calculated to be approximately 20.9% demonstrating that QC-AA@Tra has excellent phtothermal conversion efficiency. Since AIPH has been previously reported to be able to generate a large amount of thermal-dependent N_2_ and free radicals under the irradiation of 808 nm laser, we further tested the thermodynamic property of QC-AA@Tra with the hydrophilic spin trap POBN that can be used to detect free radical adducts.^21^ The ESR results (Fig. 2f) and HPLC results (Fig. S12) clearly indicate that QC-AA@Tra can stably produce carbon radicals upon the continued illumination of 808 nm laser. Together, these findings strongly support the potential of QC-AA@Tra for PTDT application.

### 3.2 Affinity test of QC-AA@Tra for HER2-positive breast cancer

**Fig. 3:**
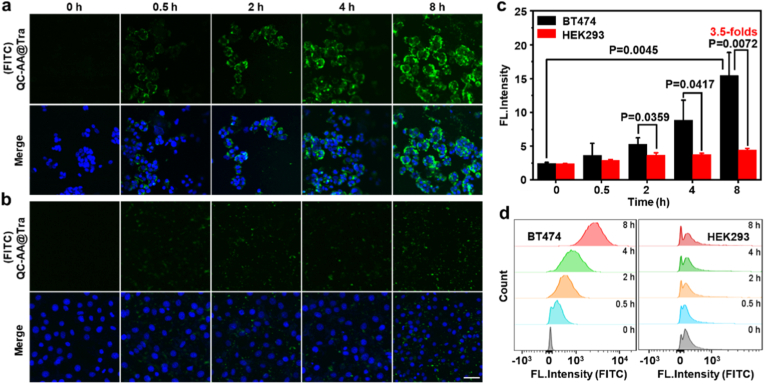
Study on targeting ability of QC-AA@Tra to BT474 breast cancer cells. Real-time FL images (a) BT474 and (b) HEK293 after co-incubation with QC-AA@Tra (1 μM). (scale bar = 50 μm) (c) Fluorescence quantification of (a) and (b). (d) Representative plots of cell populations determined by flow cytometric analysis for BT474 and HEK293 internalized by QC-AA@Tra at different incubation times. Label QC-AA@Tra with a FITC (green fluorescent) labeled secondary antibody.

To evaluate the tumor-targeting capability of QC-AA@Tra in living cells, the binding affinity of QC-AA@Tra toward intracellular HER2 receptor was first investigated as well as that of trastuzumab. The same concentration of QC-AA@Tra and trastuzumab (1 μM) was treated to breast cancer cells BT474 for 8 h followed by flow cytometry analysis. No significant difference between them was determined based on flow cytometric results (Fig. S15), implying that QC-AA@Tra possesses a similar binding affinity for HER2 receptor as natural trastuzumab. To further assess the targeting specificity of QC-AA@Tra to HER2-positive tumor cells, the same amount of QC-AA@Tra was cultured with BT474 and normal HEK293 cells, respectively, for various time points. The cells were next treated with FITC-labeled anti-trastuzumab antibody and observed under a fluorescence confocal microscope. As shown in Fig. 3a and b, strong fluorescence was distinctly detected in HER2-positive BT474 cells with 3.5 times brighter than that of HEK293 cells at 8 h (Fig. 3c), which is also validated by the flow cytometric analysis (Fig. 3d). Collectively, these results firmly demonstrate that the uptake of QC-AA@Tra in HER2-positive cancer cells is much higher than that in normal cells, revealing good targeting specificity of QC-AA@Tra to HER2-positive tumor cells.

### 3.3 PTDT of QC-AA@Tra on BT474 breast cancer cells

**Fig. 4:**
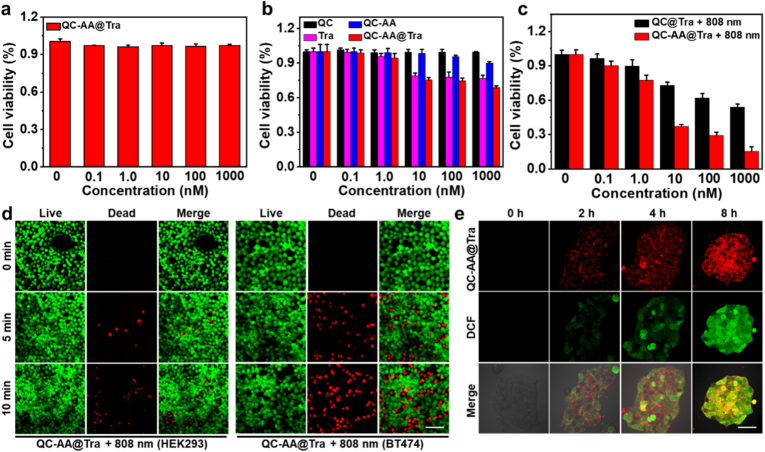
Cytotoxicity studies of the probes. (a) Cytotoxicity of various concentrations of QC-AA@Tra (0, 0.1, 1, 10, 100, and 1000 nM) to HEK293. (b) Cytotoxicities of various concentrations of QC, Tra, QC-AA, and QC-AA@Tra (0, 0.1, 1, 10, 100, and 1000 nM) to BT474. (c) Cytotoxicities of various concentrations of QC-AA@Tra (0, 0.1, 1, 10, 100, and 1000 nM) to BT474 under 808 nm laser illumination (1 W/cm^2^, 5 min). (d) FL imaging of HEK293 and BT474 cells after 24 h co-incubation of QC-AA@Tra (1 μM) followed by staining with the live/dead kit for the study of PTDT effect. Scale bar: 400 μm. (e) Detection of intracellular oxidative stress by DCFH-DA. BT474 cells were incubated with 1 μM QC-AA@Tra for 0, 2, 4, and 8 h and exposed to an 808 nm laser (1 W/cm^2^) for 5 min. Scale bar: 200 μm.

**Fig. 5:**
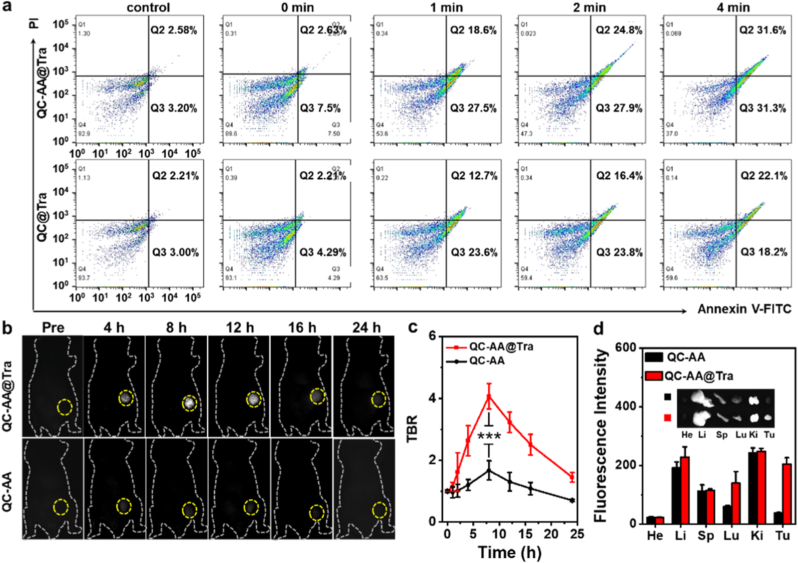
(a) Apoptosis test by flow cytometric analysis for BT474 tumors with the treatment of QC-AA@Tra (1 μM) for 8 h and exposed to an 808 nm laser (0.3 W/cm^2^). (b) Real-time NIR-II FL imaging of BT474 tumor after tail vein injection of QC-AA@Tra and QC-AA (10 mg/kg). (c) FL intensity quantization of b. (d) Fluorescence imaging and quantification of isolated organs at 8 h in b (He = heart, Li = liver, Sp = spleen, Lu = lung, Ki = kidney, and Tu = tumor). (Excitation light: 808 nm, Power: 1 mW cm^−2^, Filter: LP1000 nm).

Encouraged by great photothermal dynamic effect and tumor-targeting specificity of QC-AA@Tra, we further evaluated its inhibitory ability on cancer cell growth and thermodynamic therapeutic effect. The biocompatibility of QC-AA@Tra was first evaluated using MTT cytotoxicity assays. Different concentrations of QC-AA@Tra ranging from 0 to 1000 nM were incubated with embryonic kidney cells HEK293 for 24 h followed by CCK8 assay to detect cell viability. Fig. 4a shows that the cell viability of QC-AA@Tra is over 90% even at the concentration of 1000 nM, implying that QC-AA@Tra has negligible toxicity to normal cells. If various concentrations of QC, Tra, QC-AA, and QC-AA@Tra were incubated with BT474 cancer cells for 24 h, it was found that the cell growth could be effectively inhibited by Tra and QC-AA@Tra (Fig. 4b). This is likely due to the fact that trastuzumab specifically binds to over-expressed HER2, thereby suppressing the cell growth of BT474. We also investigated the PTDT effect of QC@Tra and QC-AA@Tra on BT474 breast cancer cells with 808 nm irradiation. Compared to QC@Tra, QC-AA@Tra shows more significant inhibition on BT474 cell growth with an IC_50_ of 5.7 nM upon 808 nm irradiation (Fig. 4c), suggesting that QC-AA@Tra has good PTDT ability. To further verify the PTDT performance of QC-AA@Tra for HER2-overexpressing tumor cells, HEK293 and BT474 cells were co-incubated with QC-AA@Tra for 24 h and illuminated by 808 nm laser for different periods of time followed by staining of the live/dead kit. Fluorescence imaging in Fig. 4d clearly indicate that the BT474 cells had more dead cells than HEK293. To examine the oxidative-stress effect of QC-AA@Tra under 808 nm irradiation, DCFH-DA as a fluorescent probe was employed to evaluate the intracellular ROS level. Fig. 4e and Fig. S16 shows that ROS level is positively correlated with the cellular uptake of QC-AA@Tra post-irradiation. Moreover, the cells treated with QC-AA@Tra and QC@Tra (5 μM) were irradiated by 808 nm laser (0.3 W/cm^2^) for various time durations and then analyzed by flow cytometry. The apoptotic rate of BT474 cells with the treatment of QC-AA@Tra was calculated to be 62.9% after 4 min irradiation, while the cells receiving QC@Tra group was only 40.3% (Fig. 5a), which should be attributed to the thermodynamic effect of encapsulated Ad-AIPH. Taken together, these findings strongly demonstrate that QC-AA@Tra is a promising multi-payload ADC for PTDT of HER2-positive cancer cells.

### 3.4 Uptake of QC-AA@Tra in BT474 orthotopic breast cancer tumors

Inspired by great PTDT effect of QC-AA@Tra on BT474 cells, we further evaluated its PTDT efficacy in vivo. The tumor imaging performance of QC-AA@Tra was first investigated in BT474 tumor-bearing mice model. QC-AA@Tra and QC-AA (10 mg/kg) were intravenously injected into two groups of mice via the tail vein. The fluorescence signals at the tumor site were then real-time monitored by IVIS imaging system. As shown in Fig. 5b, the fluorescence intensity of both groups of tumors progressively increased over time, reached a maximum at 8 h and then decayed gradually. In contrast, the accumulation of QC-AA@Tra in tumor lesion is 2.3 times that of control QC-AA according to the T/NT ratio, revealing that QC-AA@Tra has good tumor-targeting and enrichment ability (Fig. 5c). Besides, the biodistribution analysis for different organ tissues further confirmed the enrichment of QC-AA@Tra in tumors is significantly higher than that of QC-AA (Fig. 5d). Collectively, these evidences strongly demonstrate that QC-AA@Tra possesses excellent tumor specificity and has great potential for tumor treatment in vivo.

### 3.5 PTDT of BT474 orthotopic breast cancer tumors by QC-AA@Tra

**Fig. 6:**
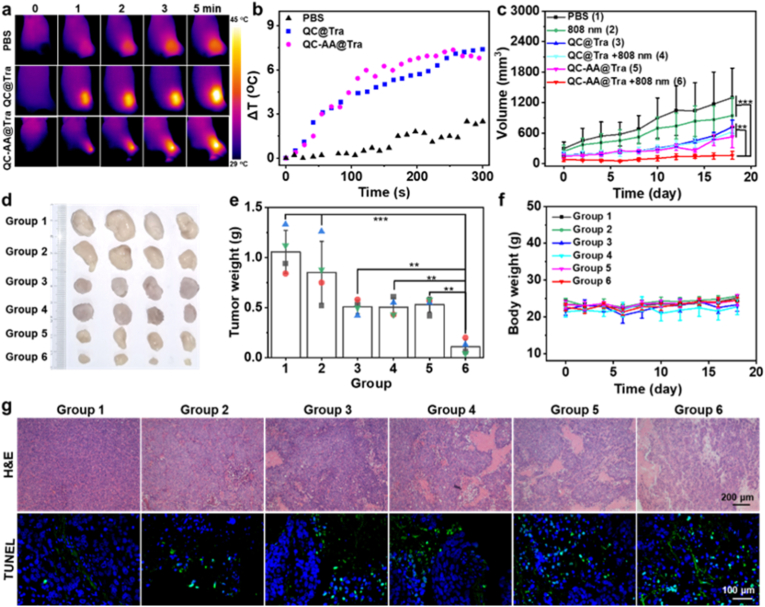
In vivo photothermodynamic therapy of BT474 breast cancers. (a) Infrared thermal images of BT474 tumors after intravenous injection of PBS, QC@Tra and QC-AA@Tra (10 mg/kg) followed by 808 nm laser irradiation (1 W cm^−2^). (b) Tumor surface temperature profiles with irradiation time in (a). (c) Relative tumor volumes for each treatment groups and (d) Images of representative tumors. (e) Relative tumor weight for each treatment group (n = 4). (f) Body weight variation of above different groups of mice. (g) H&E and Tunnel staining images of tumor slices 2 days post-treatment.

We next evaluated the PTDT efficacy on tumors. At 8 h after intravenous injection of QC-AA@Tra for BT474-bearing nude mice, the tumors were irradiated by 808 nm laser (1 W/cm^2^) for 5 min followed by temperature monitoring. Fig. 6a and b shows the local temperature of tumors in QC-AA@Tra and QC@Tra groups increased by 6.8 and 7.4°C, respectively, while that in group PBS only increased by 2.5°C, indicating that both QC-AA@Tra and QC@Tra have great photothermal effect under the irradiation of 808 nm laser. To assess the PTDT efficacy of QC-AA@Tra, six groups of BT474 tumor-bearing mice (n = 4) were subjected to different treatments (Group 1: PBS; Group 2: 808 nm; Group 3: QC@Tra; Group 4: QC@Tra+808 nm; Group 5: QC-AA@Tra; Group 6: QC-AA@Tra+808 nm). All probes were intravenously administered through the tail vein into the mice followed by 808 nm irradiation (1 W/cm^2^, 5 min) at 8 h post-injection. The tumor size of all the mice was monitored over 18 days. As shown in Fig. 6c, the tumors in groups of PBS and 808 nm display a fast growing trend in a similar manner, whereas the tumors of QC@Tra, QC@Tra+808 nm, and QC-AA@Tra were inhibited to an extent, which may be due to the chemotherapeutic effect of trastuzumab against over-expressed HER2 in tumors. In sharp contrast, the mice receiving the combination of QC-AA@Tra and 808 nm exhibited a significant tumor suppression with an inhibition rate of 87.5%. This is further validated by the resected tumors from all groups of mice (Fig. 6d and e, and S5). Additionally, no obvious weight loss was observed for all mice during the 18-day treatment period (Fig. 6f), indicating very low side effect of QC-AA@Tra on the body. Further, the H&E and TUNEL immunohistochemical staining of tumorous tissues from all groups clearly revealed that the tumors receiving QC-AA@Tra+808 nm presented severe DNA damage (Fig. 6g), implying effective therapeutic efficacy. The H&E staining of the main organs showed the mice in PBS and 808 nm groups exhibited significant lung metastasis, while no lung metastasis was determined for other groups of mice (Fig. S18). Collectively, these findings strongly demonstrate that QC-AA@Tra is a promising multi-payload ADC candidate and provides an efficient means for the treatment of HER2-positive tumors.

## 4 Conclusion and perspectives

In conclusion, we have developed a Three-site NIR-II scaffold that enables modular and multi-payload functionalization. Based on it, a novel multi-payload trastuzumab–drug conjugate QC-AA@Tra consisting of trastuzumab, NIR-II fluorophore Q3, and two Ad-AIPH-loaded cyclodextrin molecules, was successfully constructed for the treatment of HER2-positive breast cancer. Both in vitro and in vivo studies demonstrated that QC-AA@Tra exhibited excellent targeting capability toward HER2-positive BT474 tumor cells and remarkable photothermal dynamic therapeutic effect under 808 nm laser irradiation. By synergistically integrating photothermal, thermodynamic, and chemotherapeutic effects, accurate and efficient treatment of HER2-positive breast cancer was eventually achieved in living mice. This work not only validates the practical utility of this Three-site NIR-II scaffold for constructing multifunctional ADCs, but also establishes a universal strategy for developing ADCs with multiple therapeutic modalities, offering an effective tool for the precise diagnosis and treatment of HER2-positive malignant tumors.

## CRediT authorship contribution statement

**Yuqi Zhang:** Data curation, Formal analysis, Investigation, Methodology, Resources, Software, Validation, Visualization, Writing – original draft. **Yongxiang Bai:** Data curation, Methodology, Project administration, Resources, Supervision, Validation, Visualization, Writing – review & editing. **Xingxiang Ren:** Data curation, Formal analysis, Investigation, Resources, Validation, Visualization. **Yurong Fan:** Data curation, Formal analysis, Resources, Software, Validation. **Zhengzhong Lv:** Data curation, Formal analysis, Methodology, Resources, Software. **Qingfei Song:** Data curation, Formal analysis, Resources, Software, Validation. **Xiaoyan Wang:** Conceptualization, Funding acquisition, Supervision, Visualization, Writing – review & editing. **Haibin Shi:** Conceptualization, Data curation, Formal analysis, Funding acquisition, Project administration, Resources, Supervision, Validation, Visualization, Writing – review & editing.

## Declaration of competing interest

The authors declare that they have no known competing financial interests or personal relationships that could have appeared to influence the work reported in this paper.

## Data Availability

Data will be made available on request.
