# Supplementary material for: Multi-payload antibody-drug conjugates for photothermal dynamic and chemotherapy of HER2-positive breast cancer
Source: Mater Today Bio. 2026 Jun 12;39:103355. doi: 10.1016/j.mtbio.2026.103355 (PMC13285372; doi:10.1016/j.mtbio.2026.103355)
Supplement: Multimedia component 1 [file mmc1.pdf]

Supporting Information

Multi-payload Antibody-Drug Conjugates for Photothermal Dynamic and Chemotherapy of HER2-positive Breast Cancer

Yuqi Zhang,[a, #] Yongxiang Bai,[a, #] Xingxiang Ren,[a] Yurong Fan,[a] Zhengzhong Lv,[a] Qingfei Song,[b] Xiaoyan Wang,*[,b] and Haibin Shi*[,a]

[a] *State Key Laboratory of Radiation Medicine and Protection, School for Radiological and Interdisciplinary Sciences (RAD-X) and Collaborative Innovation Centre of Radiation Medicine of Jiangsu Higher Education, Soochow University, Suzhou 215123, P. R. China*

[b] *Department of Ultrasound, Heping Hospital Affiliated to Changzhi Medical College, Changzhi 046000, China*

Table of Contents

Scheme S1 2
Scheme S2 2
Figure S1 2
Figure S2 3
Figure S3 3
Figure S4 4
Figure S5 4
Figure S6 5
Figure S7 5
Figure S8 6
Figure S9 6
Figure S10 7
Figure S11 7
Figure S12 8
Figure S13 8
Figure S14 8
Figure S15 9
Figure S16 9
Figure S17 10
Figure S18 11
Figure S19 11

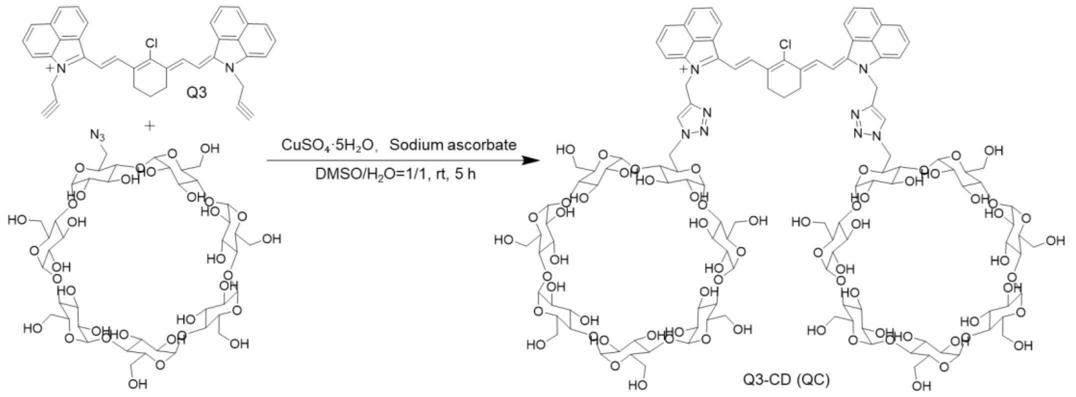

Scheme S1. Schematic representation of the synthesis procedure of Q3-CD (QC).

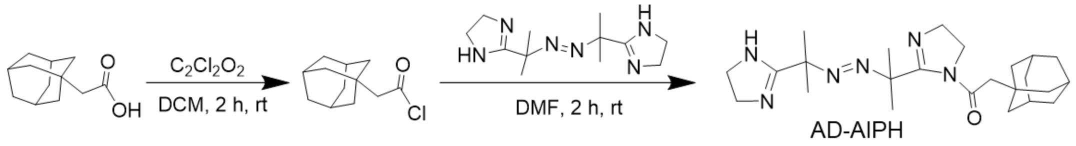

Scheme S2. Schematic representation of the synthesis procedure of Ad-AIPH.

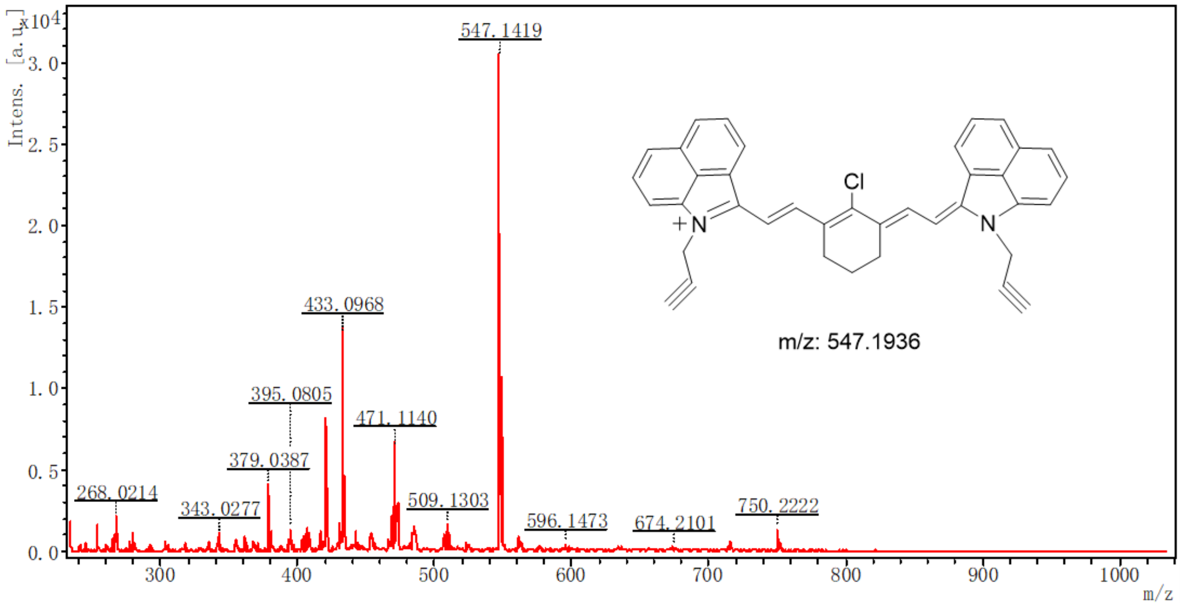

Figure S1. MALDI-TOF/MS spectrum of compound **Q3**.

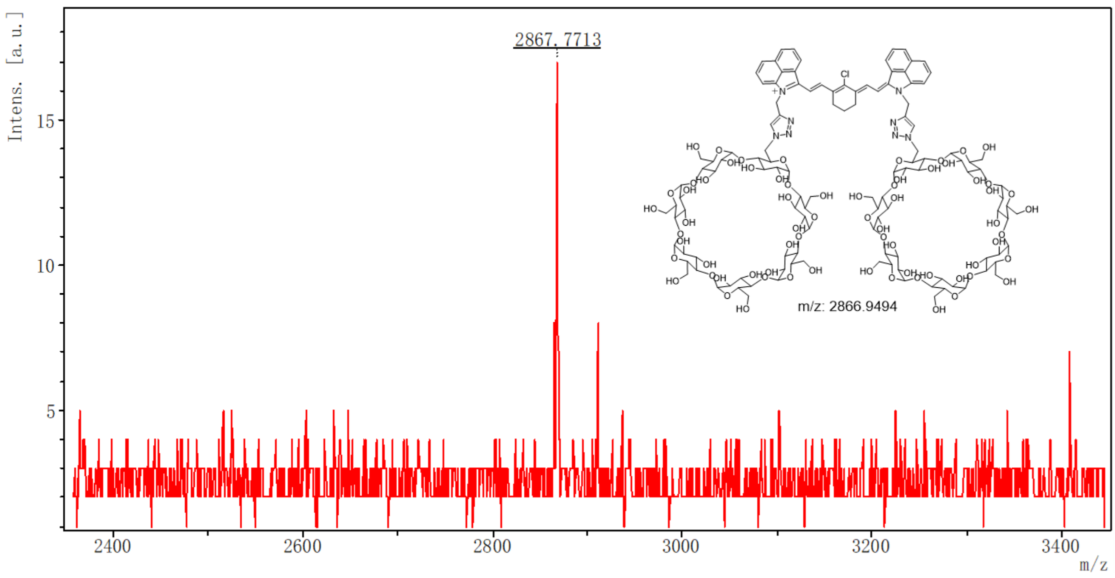

Figure S2. MALDI-TOF/MS spectrum of compound Q3-CD.

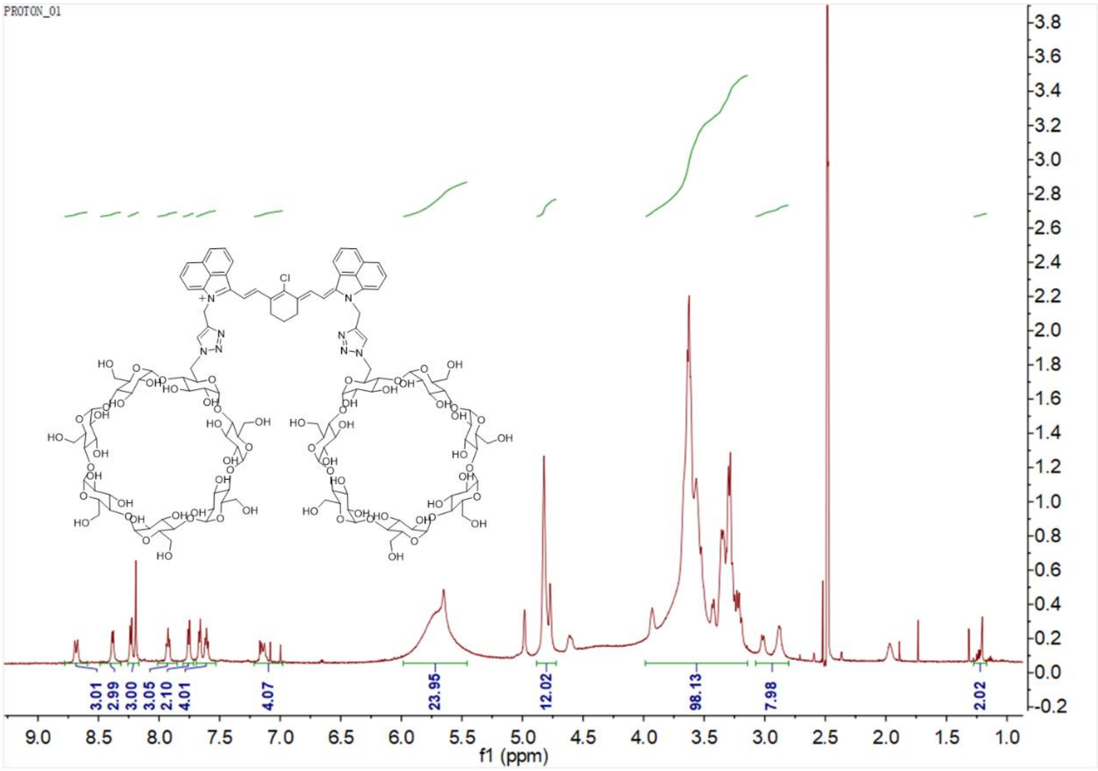

Figure S3. ^{1}H-NMR spectrum of compound Q3-CD (DMSO-d6).

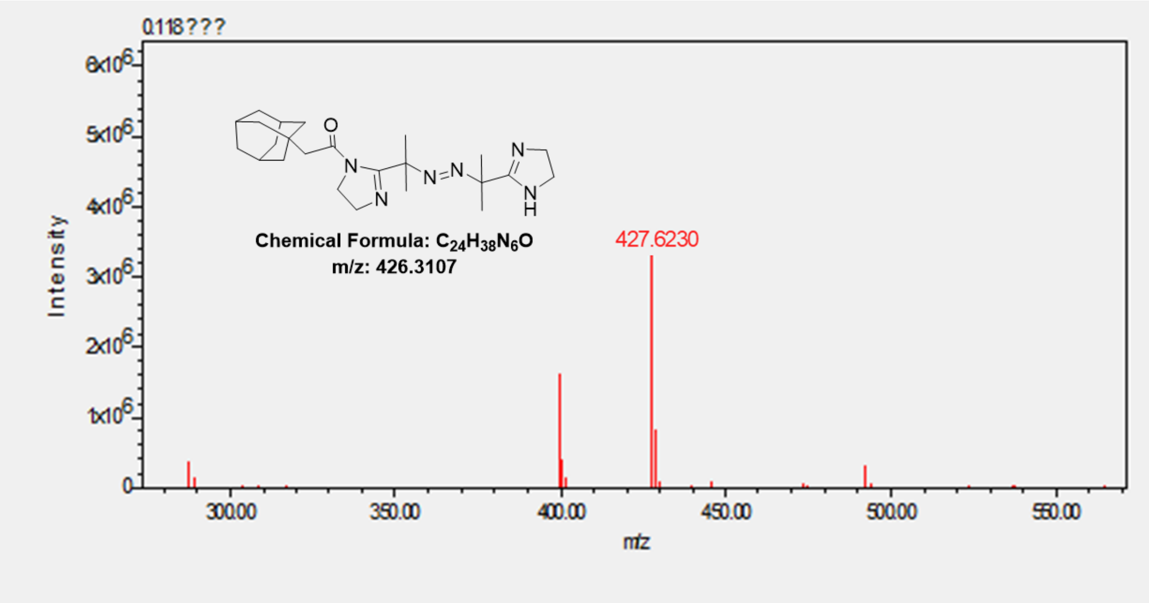

Figure S4. MALDI-TOF/MS spectrum of compound Ad-AIPH.

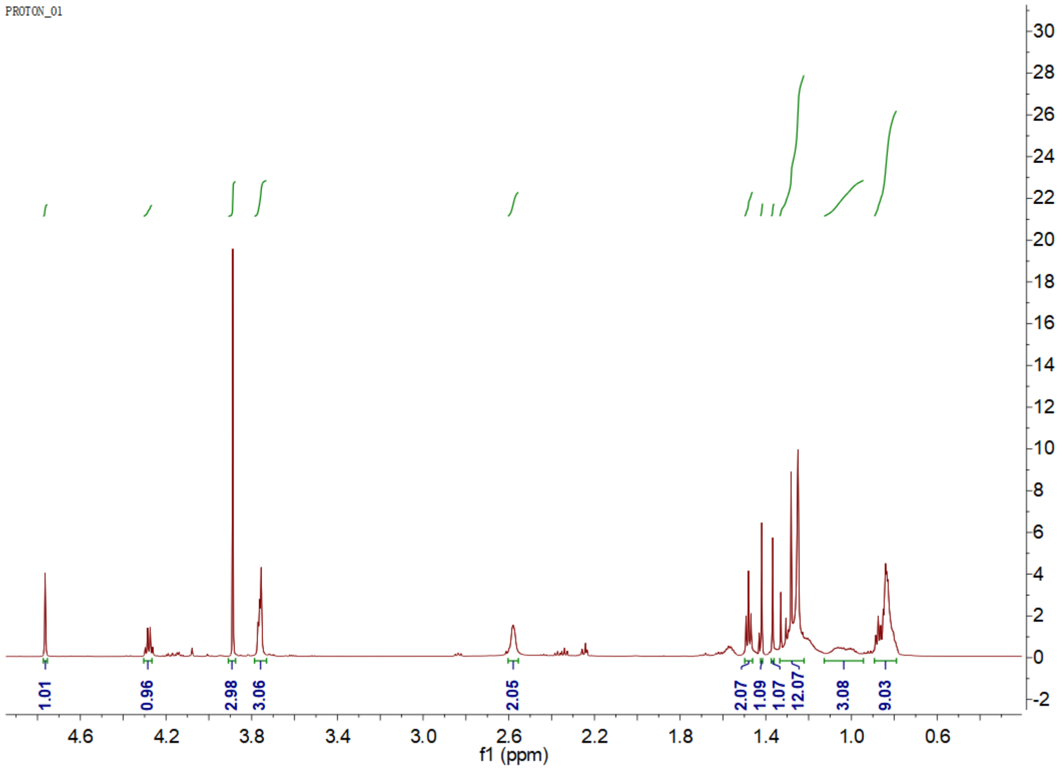

Figure S5. ¹H-NMR spectrum of compound Ad-AIPH (CDCl3).

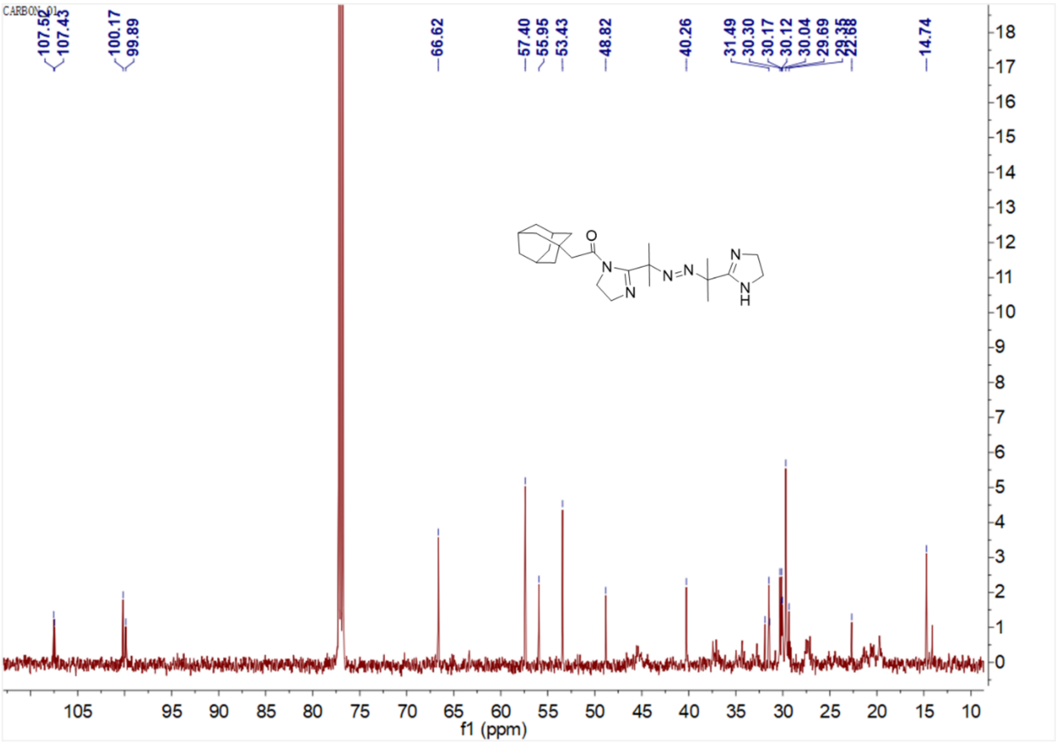

Figure S6. ^{13}C-NMR spectrum of compound Ad-AIPH (CDCl3).

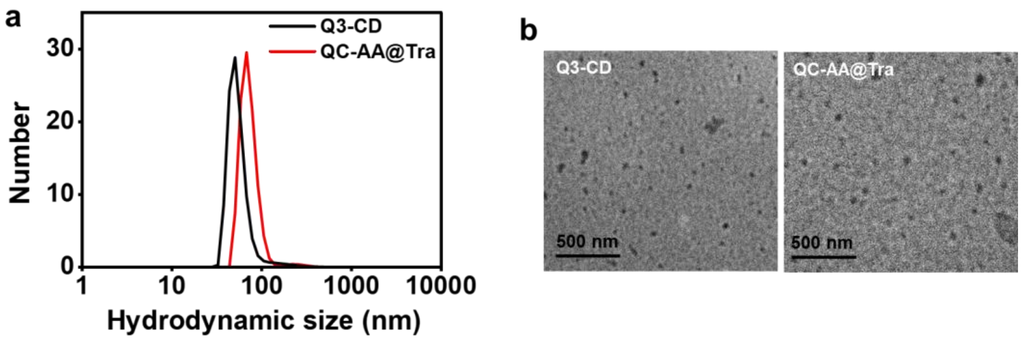

Figure S7. Dynamic light scattering (DLS) and transmission electron microscopy (TEM) characterization for both Q3-CD and QC-AA@Tra.

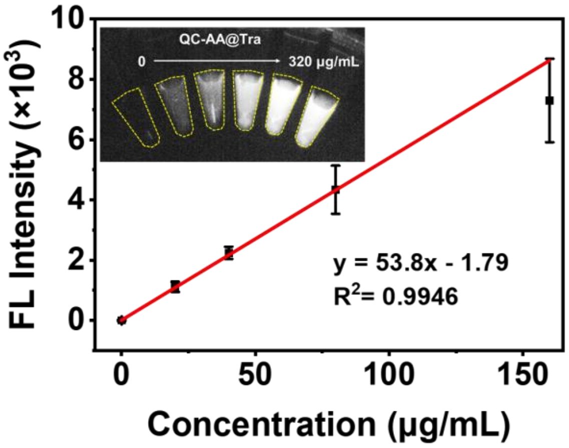

Figure S8. NIR-II fluorescence intensity and QC-AA@Tra concentration over 0-160 μg/mL.

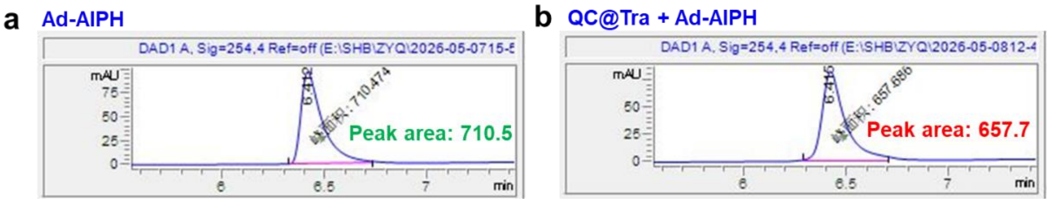

c

Encapsulation efficiency	Molar ratios (QC@Tra : Ad-AIPH)
7.4%	1 : 1.3

Figure S9. Encapsulation efficiency testing of Ad-AIPH by QC@Tra. (a) HPLC chromatogram obtained after adding 29 μg of Ad-AIPH to 100 μL of ultrapure water, sonicating for 5 minutes, centrifuging, and dissolving the precipitate in 1 mL of acetonitrile. (b) HPLC chromatogram obtained after mixing QC@Tra (34.5 μM, 100 μL) with 29 μg of Ad-AIPH, sonicating for 5 minutes, centrifuging, and dissolving the precipitate in 1 mL of acetonitrile. (c) The encapsulation efficiency of Ad-AIPH and the molar ratio of QC@Tra to Ad-AIPH, calculated from the results in (a, b) and the amounts of QC@Tra and Ad-AIPH used.

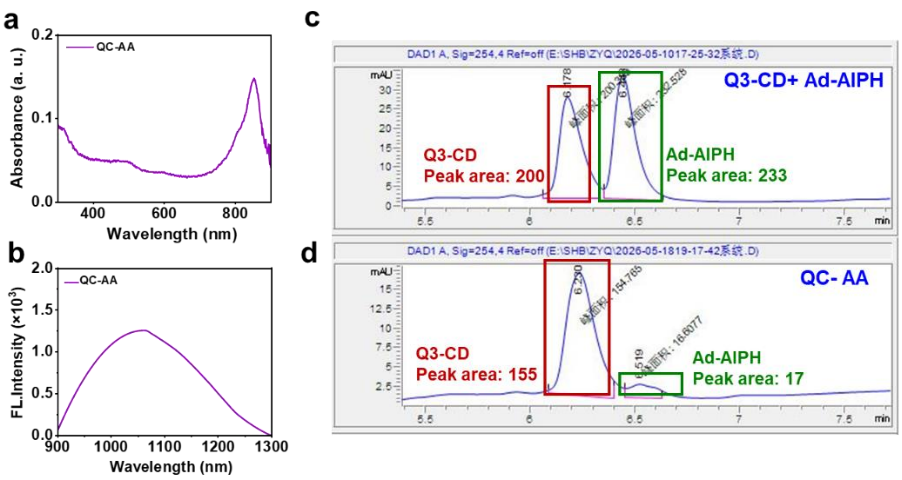

Figure S10. Optical characterization of QC-AA and encapsulation efficiency testing of Ad-AIPH. (a) UV–vis absorption spectrum of QC-AA. (b) Fluorescence spectrum of QC-AA. Encapsulation efficiency testing of Ad-AIPH using Q3-CD, (c) Q3-CD (34.5 μM, 100 μL) was mixed with 29 μg of Ad-AIPH, and the mixture was dissolved in ethanol and analyzed by HPLC. (d) The same mixture as in (c) was sonicated for 5 min, centrifuged, and the supernatant was analyzed by HPLC. Based on the results in (c) and (d), the encapsulation efficiency of Ad-AIPH was calculated to be 9.4%.

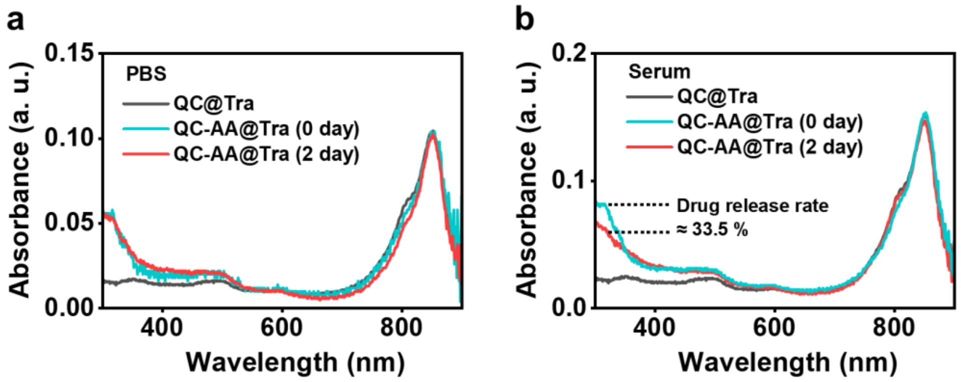

Figure S11. QC-AA@Tra was incubated in （a）PBS or （b）10% serum at room temperature for 48 hours. The retention of the loaded Ad-AIPH within QC-AA@Tra was monitored. After 48 h, approximately 33.5% of Ad-AIPH was released in 10% serum, indicating partial loss of the loaded guest under serum conditions. In contrast, no significant release was observed in PBS. These results demonstrate that QC-AA@Tra remains stable in PBS but shows moderate disassembly in the presence of serum proteins over 48 hours.

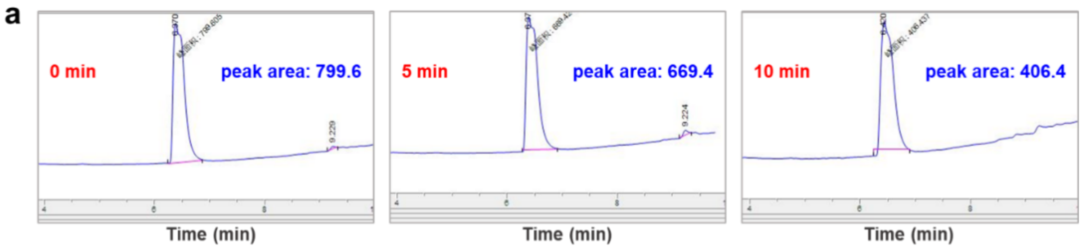

b

Time (min)	0	5	10
Degradation ratio		16.3%	49.1%

Figure S12. HPLC analysis of laser-induced degradation of Ad-AIPH in QC-AA@Tra. (a) HPLC chromatograms of QC-AA@Tra after 808 nm laser irradiation (1 W cm^{-2}) at different irradiation times (1‑10 min). (b) Time-dependent degradation percentage of Ad-AIPH under laser irradiation.

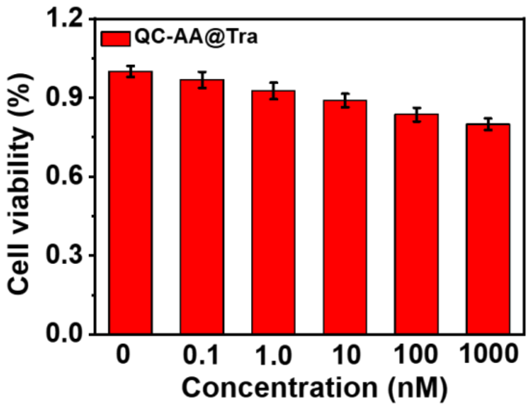

Figure S13. In vitro cytotoxicity of QC-AA@Tra against 3T3 cells. Cells were co-incubated with QC-AA@Tra for 24 h and then exposed to 808 nm laser irradiation (1 W/cm², 5 min).

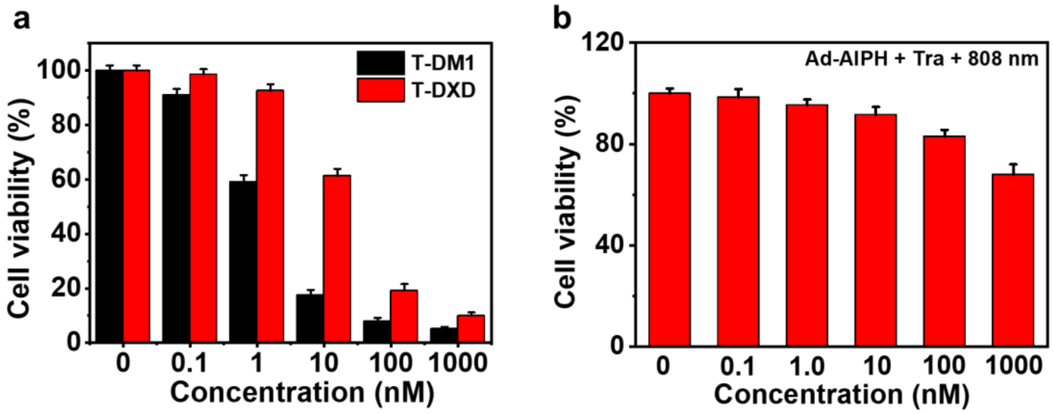

Figure S14. (a) In vitro cytotoxicity of clinical ADCs T-DM1 and T-DXd against BT474 cells; (b) Cytotoxicity of free Ad-AIPH + trastuzumab + 808 nm laser, which showed only minimal toxicity to BT474 cells.

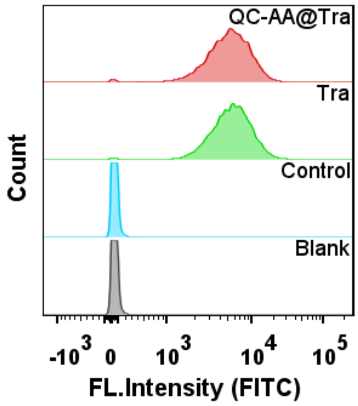

Figure S15. Representative plots of cell populations determined by flow cytometric analysis for BT474 internalized by trastuzumab (1 μM) or QC-AA@Tra (1 μM).

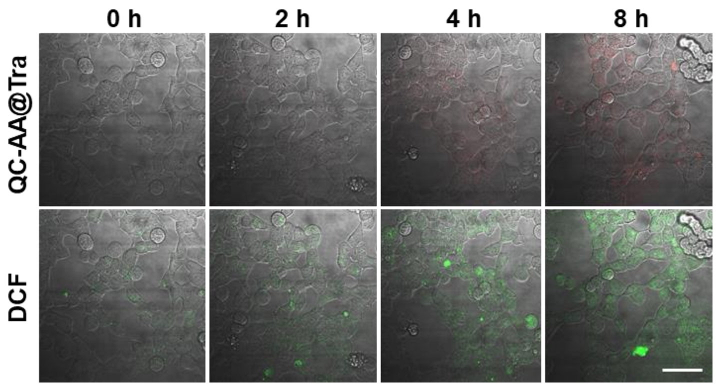

Figure S16. Detection of Intracellular Oxidative Stress by DCFH-DA. HEK293 cells were incubated with 1 μM QC-AA@Tra for 0, 2, 4, and 8 h and exposed to an 808 nm laser (1 W/cm²) for 5 min. Scale bar: 200 μm.

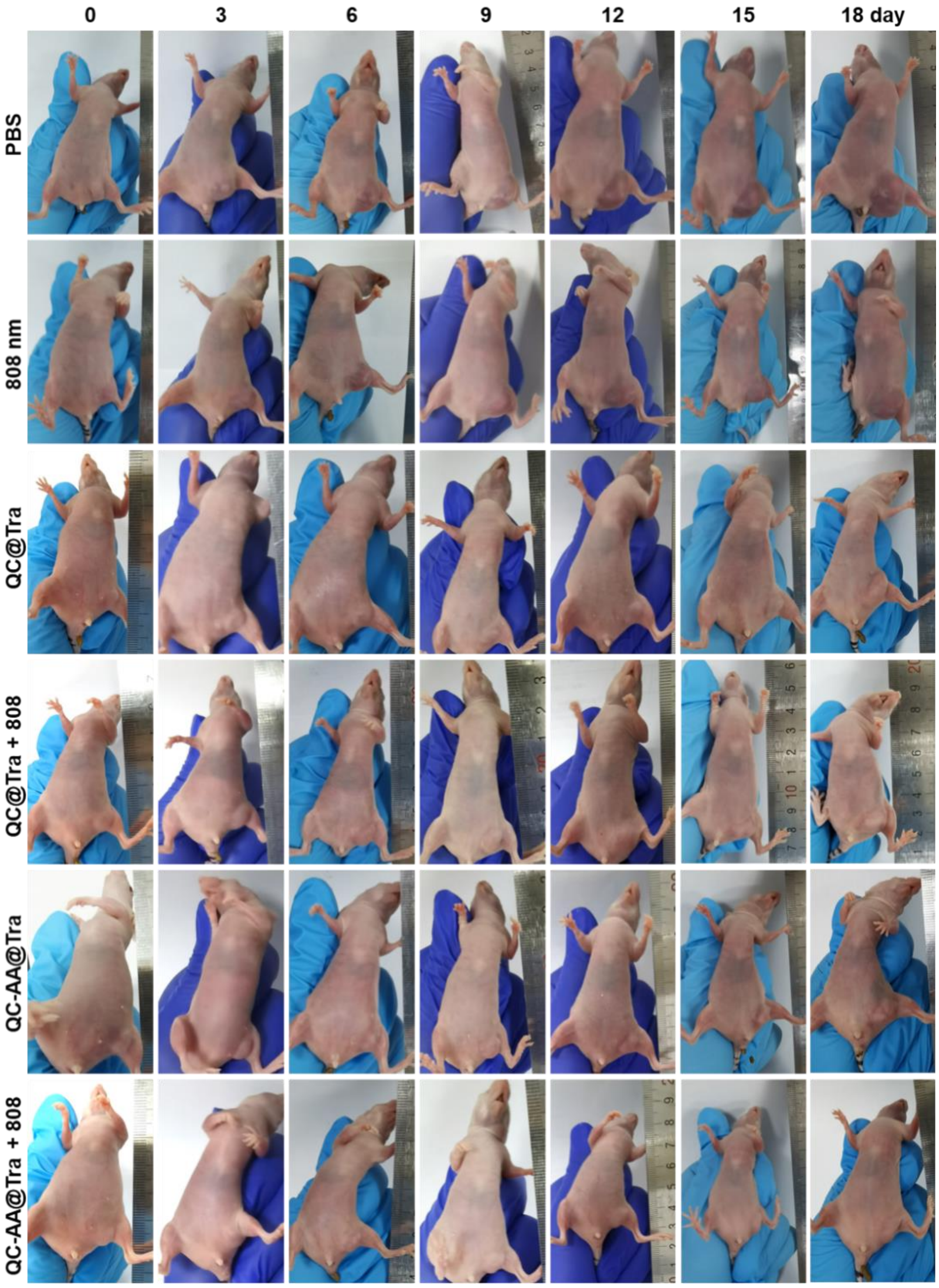

Figure S17. Images of representative mice captured on different days after each treatment.

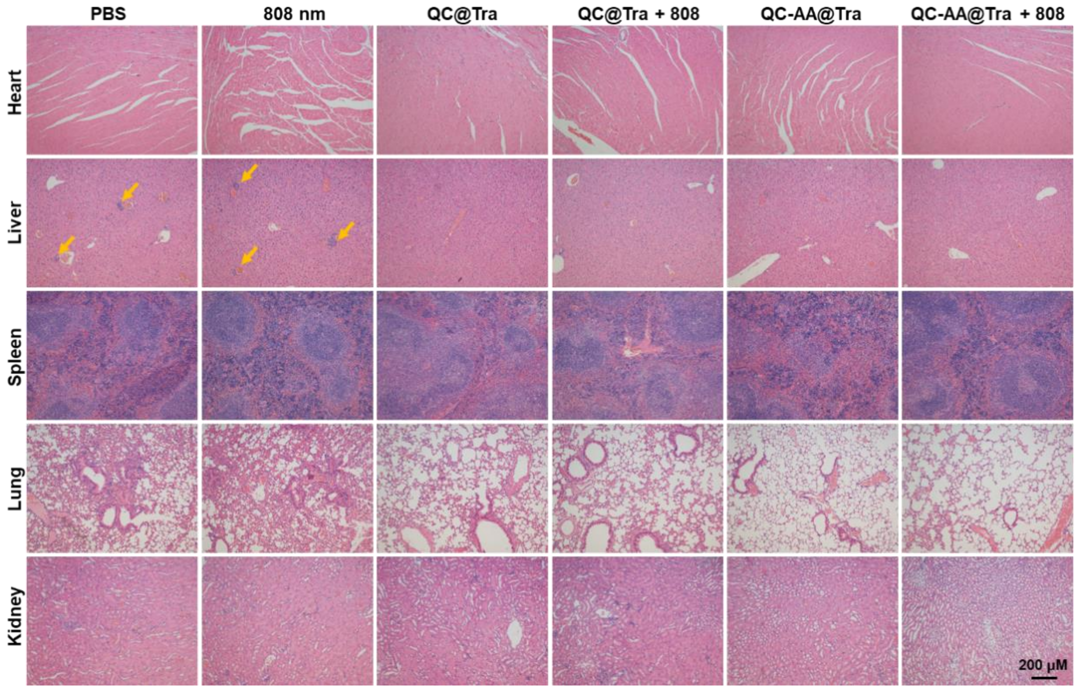

Figure S18. H&E staining of main organs from representative mice captured on 18 days after each treatment. Scale bar: 200 μm.

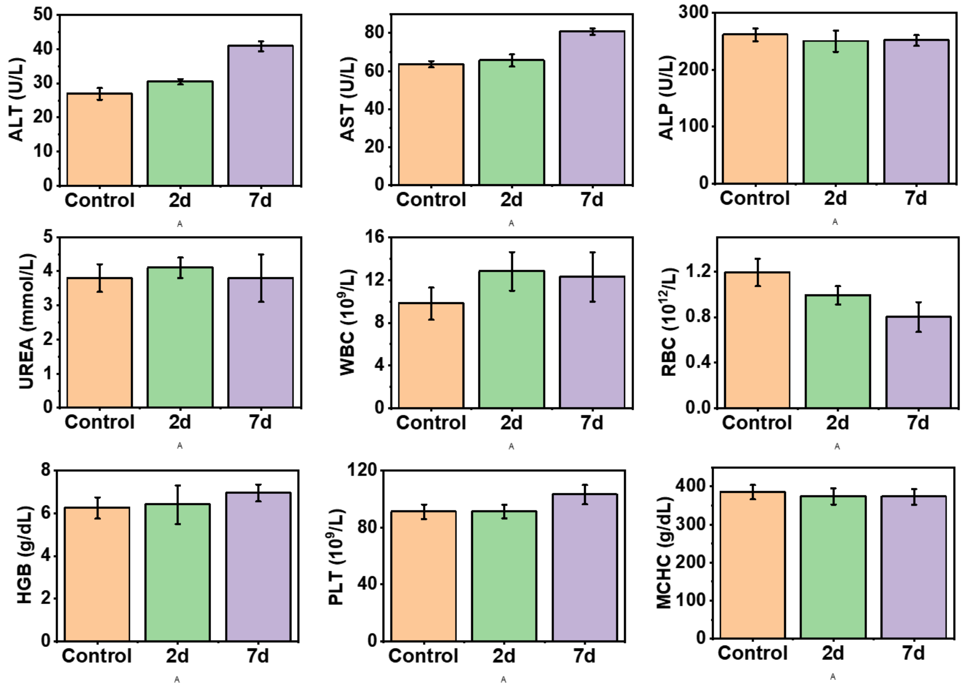

Figure S19. Variations of blood biochemical and blood routine indexes of mice with i.v. injection of QC-AA@Tra (10 mg/kg) at different time points in comparison with non-treated ones (Control). Aspartate aminotransferase, AST; alanine aminotransferase, ALT; Alkaline phosphatase, ALP; urea nitrogen, UREA; Red blood cells, RBC; White blood cells, WBC; Platelets, PLT; Mean corpuscular hemoglobin concentration, MCHC; Hemoglobin, HGB. Data are presented as mean (n = 3 independent mice).
